# Genetic and Pharmacological Inhibition of p38α Improves Locomotor Recovery after Spinal Cord Injury

**DOI:** 10.3389/fphar.2017.00072

**Published:** 2017-02-17

**Authors:** Hiroki Umezawa, Yusuke Naito, Kensuke Tanaka, Kento Yoshioka, Kenichi Suzuki, Tatsuhiko Sudo, Masahiko Hagihara, Masahiko Hatano, Koichiro Tatsumi, Yoshitoshi Kasuya

**Affiliations:** ^1^Department of Respirology, Graduate School of Medicine, Chiba UniversityChiba, Japan; ^2^Department of Biochemistry and Molecular Pharmacology, Graduate School of Medicine, Chiba UniversityChiba, Japan; ^3^Department of Biomedical Science, Graduate School of Medicine, Chiba UniversityChiba, Japan; ^4^Chemical Biology Core Facility and Antibiotics Laboratory, RIKEN Advanced Science InstituteSaitama, Japan; ^5^Corporate Research & Development, Ube Industries, LtdUbe, Japan

**Keywords:** p38 mitogen-activated protein kinases, spinal cord injury (SCI), recovery of locomotor activity, tissue degeneration, tissue regeneration

## Abstract

One of the mitogen-activated protein kinases, p38α plays a crucial role in various inflammatory diseases and apoptosis of various types of cells. In this study, we investigated the pathophysiological roles of p38α in spinal cord injury (SCI), using a mouse model. Lateral hemisection at T9 of the SC was performed in wild type (WT) and p38α^+/-^ mice (p38α^-/-^ showed embryonic lethality). p38α^+/-^ mice showed a better functional recovery from SCI-associated paralyzed hindlimbs compared to WT mice at 7 days post-injury (dpi), which remained until 28 dpi (an end time point of monitoring the behavior). In histopathological analysis at 28 dpi, there was more axonal regeneration with remyelination on the caudal side of the lesion epicenter in p38α^+/-^ mice than in WT mice. At 7 dpi, infiltration of inflammatory cells into the lesion and expression of cytokines in the lesion were reduced in p38α^+/-^ mice compared with WT mice. At the same time point, the number of apoptotic oligodendrocytes in the white matter at the caudal boarder of the lesion of p38α^+/-^ mice was lower than that of WT mice. At 14 dpi, more neural and oligodendrocyte precursor cells in the gray matter and white matter, respectively, were observed around the lesion epicenter of p38α^+/-^ mice compared with the case of WT mice. At the same time point, astrocytic scar formation was less apparent in p38α^+/-^ than in WT mice, while compaction of inflammatory immune cells associated with the wound contraction was more apparent in p38α^+/-^ than in WT mice. Furthermore, we verified the effectiveness of oral administration of SB239063, a p38α inhibitor on the hindlimb locomotor recovery after SCI. These results suggest that p38α deeply contributes to the pathogenesis of SCI and that inhibition of p38α is a beneficial strategy to recovery from SCI.

## Introduction

Spinal cord injury (SCI) results in limited motor function recovery under the chronic phase, mainly because of the poor regenerative capability of adult mammalian central nervous system (CNS) (Horner and Gage, 2000). SCI is composed of three phases, acute, secondary and chronic, and its outcomes are influenced by the secondary phase (Oyinbo, 2011). The secondary phase is characterized by inflammation-triggered events as follows: edema, apoptosis of cells including neurons and oligodendrocytes, demyelination, astrocytic scar formation and so on (Zhou et al., 2014). Under the secondary phase of SCI, gradual functional recovery is observed in several animals including humans, the extent of which is inversely related to the intensity of primary damage (Becker et al., 2003). It is thus logical to postulate that reduction of secondary damage waves by controlling inflammation-triggered events may improve the functional recovery after SCI.

p38 is one of mitogen-activated protein kinases (MAPKs) which transduces a variety of extracellular signals to the transcriptional machinery. By using genetically engineered mice, it has been demonstrated that p38 participates at least in inflammatory responses and cell fate decision including apoptosis (O’Keefe et al., 2007; Ventura et al., 2007; Kang et al., 2008; Risco et al., 2012). Among four mammalian isoforms of p38 (α, β, γ, and δ), p38α is expressed ubiquitously in adult tissues and functions as a central player of p38 isoforms (Kumar et al., 2003). Although *p38*α *gene* (*MAPK14*) deficiency results in lethality in homozygous embryonic mice, the p38α^+/-^ mouse is a useful tool for studying the *in vivo* role of p38α in certain disease models (Tamura et al., 2000; Takanami-Ohnishi et al., 2002; Matsuo et al., 2006). In particular, p38α^+/-^ mice show an outstanding resistance to neurodegenerative diseases such as epileptic seizure and experimental autoimmune encephalomyelitis (EAE, an animal model of multiple sclerosis associated with demyelination in the SC) (Namiki et al., 2007, 2012). In contrast, the deterioration of EAE was observed in transgenic mice expressing a constitutive active form of MKK6, a p38-specific activator (Noubade et al., 2011). Furthermore, self-renewal activity and neural differentiation capacity of neural stem cells (NSCs) in the hippocampus of p38α^+/-^ mice are higher than those of WT mice (Yoshioka et al., 2015). These previous findings tempt us to think that inhibition of p38α may be beneficial to the functional recovery after SCI. In fact, it has been demonstrated that a p38α inhibitor, SB203580 could reduce the damage of hindlimb function after SCI (Horiuchi et al., 2003). In contrast, another group showed that SB203580 failed to improve functional outcome after SCI (Stirling et al., 2008). Those reports are fully controversial though employing a similar SCI protocol and a same administration procedure. Therefore, whether p38α is recognized as a potential therapeutic target in SCI is still under debate.

Here, we first showed that the hindlimb locomotor behavior was improved in p38α^+/-^ mice compared to WT mice. As the mechanisms underlying the improved signs of SCI in p38α^+/-^ mice, various pathological aspects under the secondary phase of SCI were examined between WT and p38α^+/-^ mice. We finally showed that oral administration of SB239063, a p38α-specific inhibitor might be beneficial to functional recovery after SCI.

## Materials and Methods

### Animals

All animal procedures conformed to the Japanese regulations for animal care and use, following guideline for Animal Experimentation of the Japanese Association for Laboratory Animal Science, and were approved by the Animal Care and Use Committee of Chiba University. Male mice heterozygous for targeted disruption of the p38α *gene* (Tamura et al., 2000) were crossed with C57BL6J female mice (Tokyo Experimental Animal Co., Tokyo, Japan) to generate p38α^+/-^ and p38α^+/+^ [Wild type (WT)] mice. Genotyping by PCR analysis of tail-derived DNA was performed according to our previous report (Takanami-Ohnishi et al., 2002).

### SCI Model

Male WT and p38α^+/-^ mice aged 10–14 weeks were used for each experiment. Mice were deeply anesthetized with isoflurane. Laminectomy was performed at the thoracic levels of T8-10 to expose the spinal cord (SC), taking care not to damage the SC. Mice of the sham-operated group underwent laminectomy alone. Using a micro dissecting forceps, mice of the SCI group underwent right lateral hemisection at T9. Then, the muscle layer and the skin were sutured. After awaking fully from anesthesia, paralysis of the right hindlimb was assessed. The right hindlimb movement was not observed in this SCI model mice at the surgical day (day 0). At day 0, therefore, mice showing the movement of right hindlimb or with paraplegia were excluded from the following assessment and experiments. The urine was squeezed out by manual abdominal pressure on the bladder twice daily until reflex bladder function would be recovered. To determine the effect of a p38α inhibitor on SCI, each mouse received oral administration of SB239063 in acidified 0.5% tragacanth (10 mg/kg per day; Sigma–Aldrich, St. Louis, MO, USA) at 1, 2, and 3 dpi, and the control group received oral administration of vehicles, acidified 0.5% tragacanth.

### Behavior Study

We evaluated the motor function of the SCI-associated paralyzed hindlimbs from 0 to 28 days post-injury (dpi), based on Basso Mouse Scale (BMS) (Basso et al., 2006). The BMS is a 9 point scale for assessment of functional recovery of mice’s hindlimbs. Mice were forced to walk in an open field, and their right hindlimbs movement was observed for 4 min to score based on BMS.

### Tissue Preparation and Histological Analysis

Mice were anesthetized lethally and transcardially perfused with ice-cold phosphate-buffered saline (PBS). SCs including the lesions were carefully dissected out, fixed overnight in 4% paraformaldehyde and subsequently immersed in 30% sucrose for 2 days to cryoprotect the tissues. After embedding into OCT compound, the samples were transversely or sagittally sectioned at a thickness of 20 μm. Sagittal and transverse sections were stained with hematoxylin-eosin (HE) and by a Kluver-Barrera’s (KB) method, respectively. The injury-associated leukocyte infiltration area and Luxol Fast Blue (LFB)-staining area in sections stained with HE and by a KB method, respectively, were quantified using Macromax MVC-DU (GOKO, Kanagawa, Japan).

### Tracing Study

For anterograde tracing of axons, a total of 2 μl of 10% Texas Red-conjugated biotinylated dextran amine (Texas Red-BDA; Vector Laboratories, Burlingame, CA, USA) was injected into four sites of sensorimotor cortex in the left side (contralateral to the side of injured SC) at 14 dpi. The scalp was cut and a hole was carefully drilled into the skull, and then Texas Red-BDA was injected into the sensorimotor cortex using a 10 μl Hamilton microsyringe. The scalp was closed with suture. Mice applied with Texas Red-BDA were killed at 28 dpi. Sagittal sections (20 μm thick) from the SCs were observed by a fluorescence microscope (Axio Imager A2, Zeiss, Oberkochen, Germany). Texas Red-BDA-staining area in a visual field was quantified using ImageJ 1.45.

### Immunofluorescence Study

The freshly cut sagittal sections (20 μm thick) placed on poly-L-lysine-coated slides were pretreated with 1:10 FcR blocking agent (Miltenyi Biotec, Gladbach, Germany) for 10 min and reacted with various primary antibodies as follows: anti-CNPase (Sigma–Aldrich, St. Louis, MO, USA) to label oligodendrocytes, anti-cleaved caspase-3 (Cell Signaling Tech., Beverly, MA, USA) to label apoptotic cells, Cy3-conjugated anti-glial fibrillary acidic protein (GFAP; Sigma–Aldrich) to label astrocytes, anti-Iba1 (WAKO, Osaka, Japan) to label microglia or macrophages, Cy3-conjugated anti-NG2 (Merck Millipore, Billerica, MA, USA) to label to oligodendrocyte precursors, FITC-conjugated anti-CD45.2 (BioLegend, San Diego, CA, USA) to label leukocytes, biotin-labeled anti-CD3 (Affymetrix, Santa Clara, CA, USA) to label T lymphocytes. After staining with each appropriate fluorescein-conjugated second antibody or streptavidin, 4′, 6-diamidino-2-phenylindole (DAPI) was applied for nuclear staining before the final washing step. The sections were observed by a fluorescence microscope. In case of counting cells immunoreacted with antibodies, 4–5 sections from each SC were randomly selected. Under 200× magnification, two fields within 1 mm centered on the lesion epicenter in the SCI group or the corresponding segment in the sham-operated group were randomly chosen in each section, and fluorescent signal-expressing cells were counted and averaged (/0.1 mm^2^).

### Western Blot Array Analysis

Male WT and p38α^+/-^ mice of the sham-operated and SCI groups were anesthetized and sacrificed at 7 dpi. Then, each unilateral SC in the right side including with the injured region was dissected out, and cut with the length of 6 mm from the edge of the rostral lesion to caudal side. The SC sample was homogenized, and centrifuged at 9000 × *g* for 20 min at 4°C. The resulting supernatant was subjected to protein assay. Protein sample (60 μg/mouse) from five mice of each group (WT-sham, WT-SCI, p38α^+/-^-sham, p38α^+/-^-SCI) was mixed (300 μg in each group) and subjected to RayBio^®^ Biotin Label-based Mouse Antibody Array 1 (RayBiotech, Norcross, GA, USA), and changes in expression levels of 308 inflammation-related proteins in the samples were evaluated. The array was performed according to the manufacturer’s instructions. Using a densitometer, each signal was normalized to the positive internal controls included in the array membrane and expressed as induction ratio of the sham-operated value.

### Statistical Analysis

All analyses were conducted through GraphPad Prism Version 6 (GraphPad Software, San Diego, CA, USA). Statistical significance was determined by Mann–Whitney *U* test, Student’s *t*-test or analysis of variance (ANOVA) followed by Tukey’s test, and *P*-value of < 0.05 were considered to be significant.

## Results and Discussion

### Recovery of Hindlimb Locomotor Behavior Related to Histopathological Findings in SC after SCI between p38α^+/-^ and WT Mice

**FIGURE 1:**
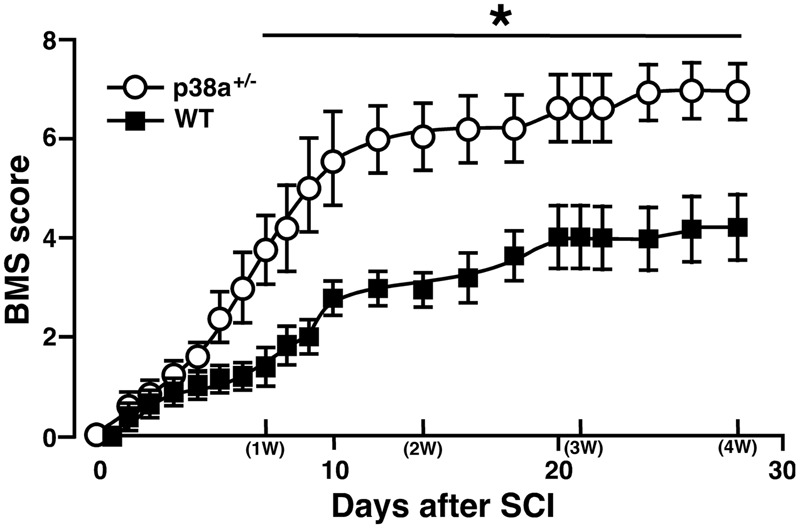
**Time course of hindlimb locomotor behavior after spinal cord injury (SCI).** Wild type (WT) and p38α^+/-^ mice with SCI were observed daily until 28 dpi, and scored based on BMS. Data are shown as mean ± SEM (*n* = 5). The difference between WT (filled squares) and p38α^+/-^ (open circles) mice was statistically significant (^∗^*P* < 0.05) as determined by Mann–Whitney *U* test for unpaired values at each time point.

We first addressed to whether a single copy disruption of *p38*α *gene* might affect functional recovery of hindlimb after lateral hemisection employed as SCI model in this study. Although such a laceration injury of SC is not typically seen clinically, a hemisection model is suitable to investigate the pathophysiological elements inhibiting or promoting axonal regeneration across or around the laceration injury as well as the resulting functional impairment and potential recovery (Onifer et al., 2007). As shown in **Figure 1**, p38α^+/-^ mice showed significantly less severe neurological function of paralyzed SCI-associated right hindlimbs at 7 dpi, and then the more improved locomotor function in p38α^+/-^ mice than in WT mice remained until 28 dpi. Therefore, a single copy disruption of *p38*α *gene* suppressed the functional disturbance in the hemisection model of SCI.

**FIGURE 2:**
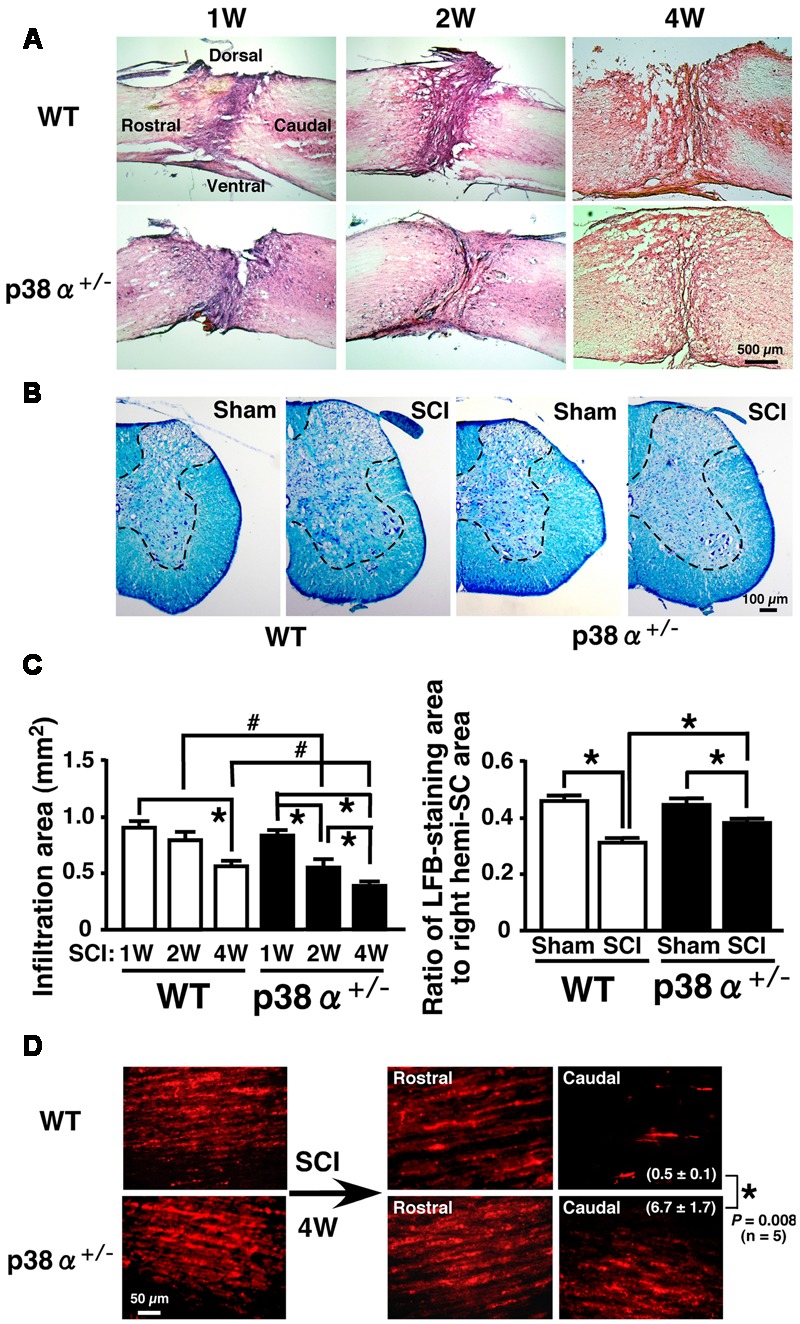
**Histopathological changes after SCI. (A)** Typical profiles of HE-stained sagittal SC sections from WT and p38α^+/-^ mice at 1, 2, and 4 weeks post-injury. **(B)** Transverse SC sections from WT and p38α^+/-^ mice at 4 weeks post-injury (1 mm caudal of the lesion epicenter) or with sham-operation (the corresponding site) were stained by a KB method. LFB-staining area was surrounded with the dotted lines. **(C)** Quantitative evaluation of **(A)** and **(B)**. Left column, SCI-associated leukocyte infiltration into SC of WT (open bars) and p38α^+/-^ (closed bars) mice. Right column, ratio of LFB-staining area to right hemi-SC area in WT (open bars) and p38α^+/-^ (closed bars) mice. Data are shown as mean ± SEM (*n* = 5). ^∗^*P* < 0.05 (ANOVA followed by Tukey’s test), ^#^*P* < 0.05 (Student’s *t*-test for unpaired values). **(D)** Representative images of Texas Red-BDA-labeled axons in the white matter of SC of WT and p38α^+/-^ mice at 4 weeks post-injury. In the SCI group, sagittal sections of SC at 5 mm rostral and 5 mm caudal to the lesion epicenter were observed. In the sham-operated group, SC segment corresponding to the lesion epicenter (T9) of the SCI group was observed. Texas-Red BDA staining area caudal to the lesion epicenter was expressed as the percentage of the whole field. Data are shown as mean ± SEM (*n* = 5). ^∗^*P* < 0.05 (Student’s *t*-test for unpaired values).

Then, histopathological changes such as leukocytic infiltration-associated lesion area, myelinated area and axonal regeneration between the two genotypes were examined after SCI (**Figure 2**). We calculated the leucocytic infiltration area using sagittal sections stained with HE at 1, 2, and 4 wpi (**Figures 2A,C**). There was no significant difference in the size of leukocyte infiltration-associated lesion between the two genotypes at 1 wpi. The SCI-induced lesion was reduced in a time-dependent manner in the two genotypes at 2 and 4 wpi but significantly smaller in p38α^+/-^ mice than in WT mice at each time point. These results suggest that the functional and histopathological recovery after SCI may be enhanced or accelerated in p38α^+/-^ mice compared with WT mice. Remyelination of regenerated axons are likely to be one of key mechanisms involved in the spontaneous recovery of motor function after SCI (Lu et al., 2012). As shown in **Figure 2B**, Luxol Fast Blue (LFB)-stained area on transverse section of the SC of the two genotypes was significantly smaller in the SCI group compared with the sham-operated group. And, the LFB-positive ratio was significantly larger in p38α^+/-^ mice than in WT mice at 4 wpi (**Figure 2C**). At 2 wpi, the LFB-positive staining in the white matter of SC of the two genotypes was much weaker than the case at 4 wpi (data not shown), suggesting that axonal remyelination may occur at least over a time period ranging from 2 to 4 wpi. Likewise, axons labeled by an anterograde tracer, Texas Red-BDA were more frequently observed in caudal part of the SC of p38α^+/-^ mice than of WT mice at 4 wpi, a semi-quantitative analysis of which showed a significant difference between the both genotypes (**Figure 2D**). The labeled frequency in rostral part of the SC was equally high between the two genotypes because axonal degeneration predominantly occurred in caudal part of the SC, and axons are intact in 5 mm rostral to the lesion epicenter of SC after the hemi-section injury. These results suggest that axonal regeneration and remyelination after SCI may be enhanced or accelerated in p38α^+/-^ mice compared with WT mice. To elucidate the mechanism underlying the improved signs of SCI in p38α^+/-^ mice, we focused on various pathological events at 1 and 2 wpi in which the difference in improvement of spontaneous locomotor ability after SCI between p38α^+/-^ mice and WT mice was recognized and then manifested.

### Characterization of Inflammatory Response and Evaluation of Oligodendrocyte Apoptosis between p38α^+/-^ and WT Mice at 1 wpi

**FIGURE 3:**
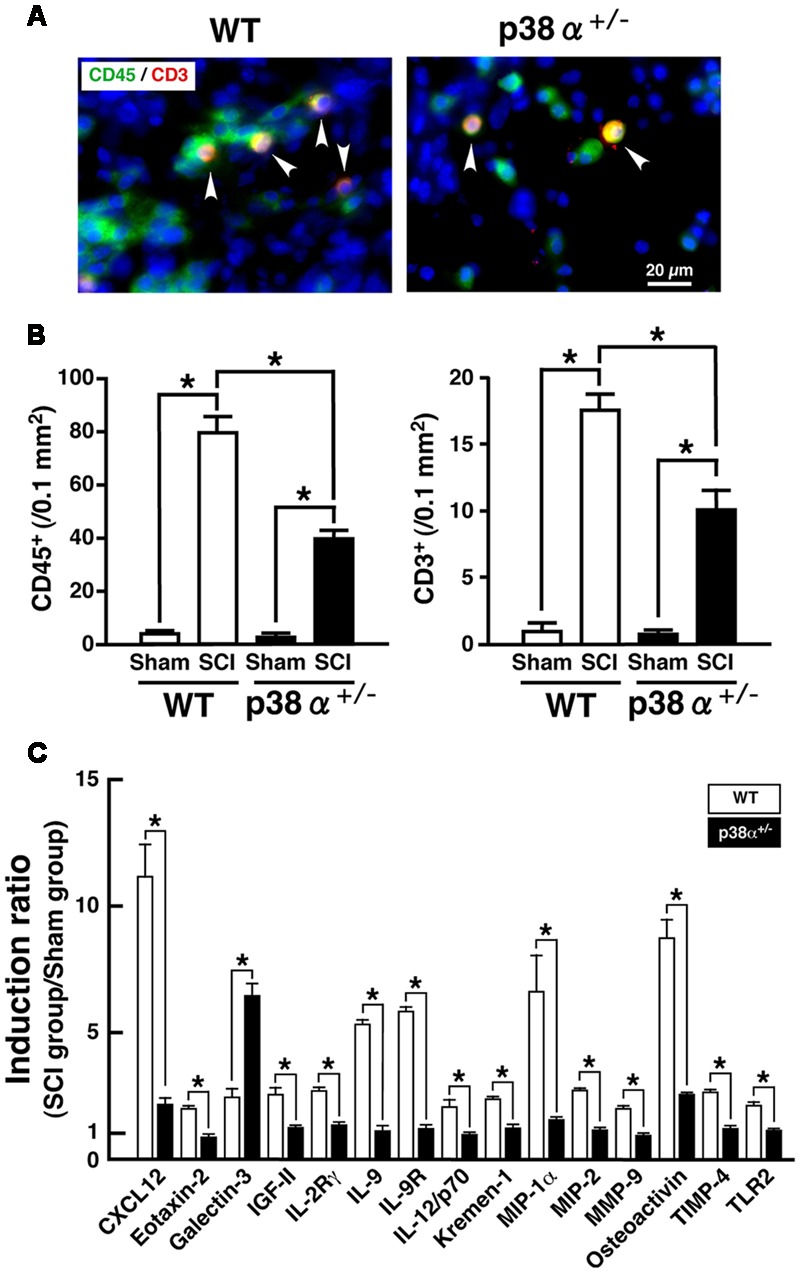
**Inflammatory profiles 1 week after SCI. (A)** Typical profile of CD3^+^CD45^+^ cells in the lesion epicenter of the two genotypes at 1 week post-injury. Asterisks indicate double-positive cells. **(B)** Quantitative evaluation of **(A)**. CD3^+^ cells and CD45^+^ cells in the injured site within 500 μm rostrocaudal of the lesion epicenter increased in both WT (open bars) and p38α^+/-^ (closed bars) mice at 1 week post-injury, the number of which was significantly lower in p38α^+/-^ mice than WT mice. Data are shown as mean ± SEM (*n* = 5). ^∗^*P* < 0.05 (ANOVA followed by Tukey’s test). **(C)** SCI-induced changes in expression of cytokines in the SC between WT and p38α^+/-^ mice. Collected SC protein sample from five mice of each group (WT-sham, WT-SCI, p38α^+/-^-sham or p38α^+/-^-SCI) were subjected to protein array for 308 molecules. Three independent experiments were conducted (15 mice in each group). Using a densitometer, each signal was normalized to the positive internal controls included in the array membrane (P1-a), and expressed as induction ratio of the sham-operated value. Among 61 molecules (≥2, induction ratio in WT group), 15 molecules showed a significant difference in their induction ratio between the two genotypes. Data are shown as mean ± SEM (*n* = 3). The difference between WT (open squares) and p38α^+/-^ (filled squares) mice was statistically significant (^∗^*P* < 0.05) as determined by Student’s *t*-test for unpaired values.

A significant importance of leukocytes-mediated inflammatory reaction is well known in the development of SCI. In particular, T lymphocytes invade the lesion site, concomitantly to macrophages and secrete cytokines in the lesion epicenter, which results in axonal damage and motor neuron apoptosis after SCI (Brunn et al., 2008; Beck et al., 2010). Moreover, a previous report demonstrated that the expression of p38 was enhanced in resident and infiltrating immune cell after SCI (Stirling et al., 2008). Thus, we elucidated cell populations of leukocytes and T lymphocytes detected as CD45^+^ and CD3^+^, respectively, in the lesion. As shown in **Figures 3A,B**, CD45^+^ and CD3^+^ cells were observed in the lesion of the two genotypes at 1 wpi, each number of which was significantly lower in p38α^+/-^ mice than WT mice. In general, neutrophils are a major cell population of CD45^+^ cells and contribute to both the progression of damage and the tissue repair after SCI (Neirinckx et al., 2014). However, Ly6G^+^ neutrophils were hardly detected in the lesion at 1 wpi (data not shown), which was supported by a previous report that neutrophil recruitment showed fast kinetics reaching a peak at 1 day and immediately declining to the baseline within several days (Donnelly and Popovich, 2008). Considering cell types of leukocytes in the inflammation of SCI, thus, CD45^+^CD3^-^ cells in the lesion at 1 wpi may be mainly monocytes/macrophages. At the same time point, we also investigated the comprehensive analysis of SCI-induced change in expression of inflammation-related proteins in the SC. As shown in **Figure 3C**, we found 15 molecules [C-X-C motif chemokine 12 (CXCL12); Eotaxin-2; Galectin-3; insulin-like growth factor 2 (IGF-II), IL-2 receptor γ (IL-2Rγ); IL-9; IL-9 receptor (IL-9R); IL-12/p70; Kremen-1; macrophage inflammatory protein 1α (MIP-1α); MIP-2; matrix metalloproteinase 9 (MMP-9); Osteoactivin; tissue inhibitors of metalloproteinase 4 (TIMP-4); Toll-like receptor 2 (TLR2)] showing a clear difference in their expression between WT and p38α^+/-^ mice. The lower expression of MIP-1α, MIP-2, and MMP-9 was corroborated as less leucocyte infiltration in the injured SC of p38α^+/-^ mice (Jaerve and Müller, 2012). In particular, MMP-9 has been thought to open the blood-SC barrier and promote migration of leukocytes into the lesion, which may directly influence the severity of SCI (Noble et al., 2002). It has been also reported that MMP-9 and CXCL12 function synergistically to facilitate migration of blood-borne monocyte (Zhang et al., 2011), although CXCL12 has been regarded as one of key chemoattractants that regulates migration of homeostatic stem and progenitor cells in animal models of CNS injury and promote axonal sprouting (Jaerve and Müller, 2012). Therefore, it can be speculated that the decreased expression of both MMP-9 and CXCL12 in p38α^+/-^ mice may lead to the less infiltrating leucocytes (Stirling et al., 2008). Furthermore, MIP-1α and CXCL12 can recruit T lymphocytes into the injured site (Ousman and David, 2001; Jaerve and Müller, 2012). Thus, their decreased expression may be closely related to the reduction of T cell infiltration in p38α^+/-^ mice. On the other hand, Galectin-3 and TLR2 have been reported to have protective effects on SCI through regulating inflammatory response (Kigerl et al., 2007; Stirling et al., 2014; Gensel et al., 2015; Mostacada et al., 2015). Among the 15 molecules, only SCI-induced Galectin-3 was higher in p38α^+/-^ mice compared with WT mice, suggesting that the increase of Galectin-3 may also contribute to the less infiltrating leucocytes in p38α^+/-^ mice. Further study is needed to elucidate whether functional inhibition of each molecule by its neutralizing antibody affects the severity of SCI. However, the decrease in concomitant infiltration of monocytes/macrophages and T lymphocytes associated with the changes in expression of several cytokines/chemokines may contribute to the less development of SCI in p38α^+/-^ mice.

**FIGURE 4:**
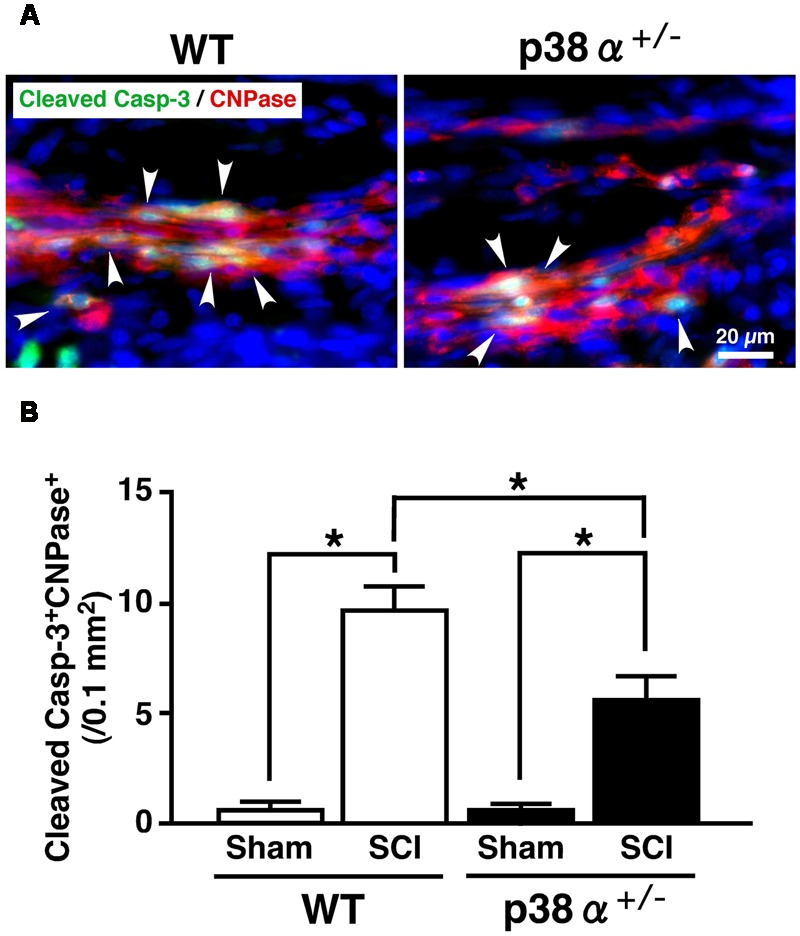
**Changes in numbers of apoptotic oligodendrocytes in the white matter 1 week after SCI. (A)** Typical profile of cleaved caspase-3^+^CNPase^+^ cells in the white matter at the caudal boarder of the lesion of the two genotypes at 1 week post-injury. Asterisks indicate double-positive cells. **(B)** Cleaved caspase-3^+^CNPase^+^ cells in the injured site within 500 μm rostrocaudal of the lesion epicenter increased in both WT (open bars) and p38α^+/-^ (closed bars) mice at 1 week post-injury, the number of which was significantly lower in p38α^+/-^ mice than WT mice. Data are shown as mean ± SEM (*n* = 4–5). ^∗^*P* < 0.05 (ANOVA followed by Tukey’s test).

In CNS, p38 mainly localizes in myelin sheath but not in axon (Maruyama et al., 2000). It has been reported that inhibition of p38 prevents myelin structure destruction associated with oligodendrocytic apoptosis and ameliorates neurological deficits after SCI (Horiuchi et al., 2003). It has also been reported that apoptosis of oligodendrocyte would occur about 1 week after SCI and cause demyelination and axonal disturbance (Li et al., 1999; Dong et al., 2003). As a reliable strategy, colocalized cells of CNPase and cleaved caspase-3 are regarded as oligodendrocytes under programmed cell death in human SCI (Emery et al., 1998). Then, we evaluated a cell population of cleaved caspase-3^+^CNPase^+^ cells in the white matter of the lesion. As shown in **Figure 4**, cleaved caspase-3^+^CNPase^+^ cells were typically observed in the SCI group of the two genotypes at 1 wpi (A), the number of which was significantly lower in p38α^+/-^ mice than WT mice (B). On the other hand, Nissle^+^ neuronal cells decreased in the gray matter of SC around the lesion epicenter in the two genotypes at 1 wpi, which was significantly moderate in p38α^+/-^ mice than WT mice (Supplementary Figure 1). These results indicated that p38α^+/-^ mice showed resistance to cell death of oligodendrocytes and neurons at 1 wpi. Hence, the inflammatory responses and cell death of oligodendrocytes and neurons after SCI were reduced by a single copy disruption of *p38*α *gene*. Next, we elucidated a role of p38α in the tissue regeneration process after SCI.

### Astrocytic Scar Formation and Increase in Oligodendrocyte Precursor Cells after SCI between p38α^+/-^ and WT Mice

**FIGURE 5:**
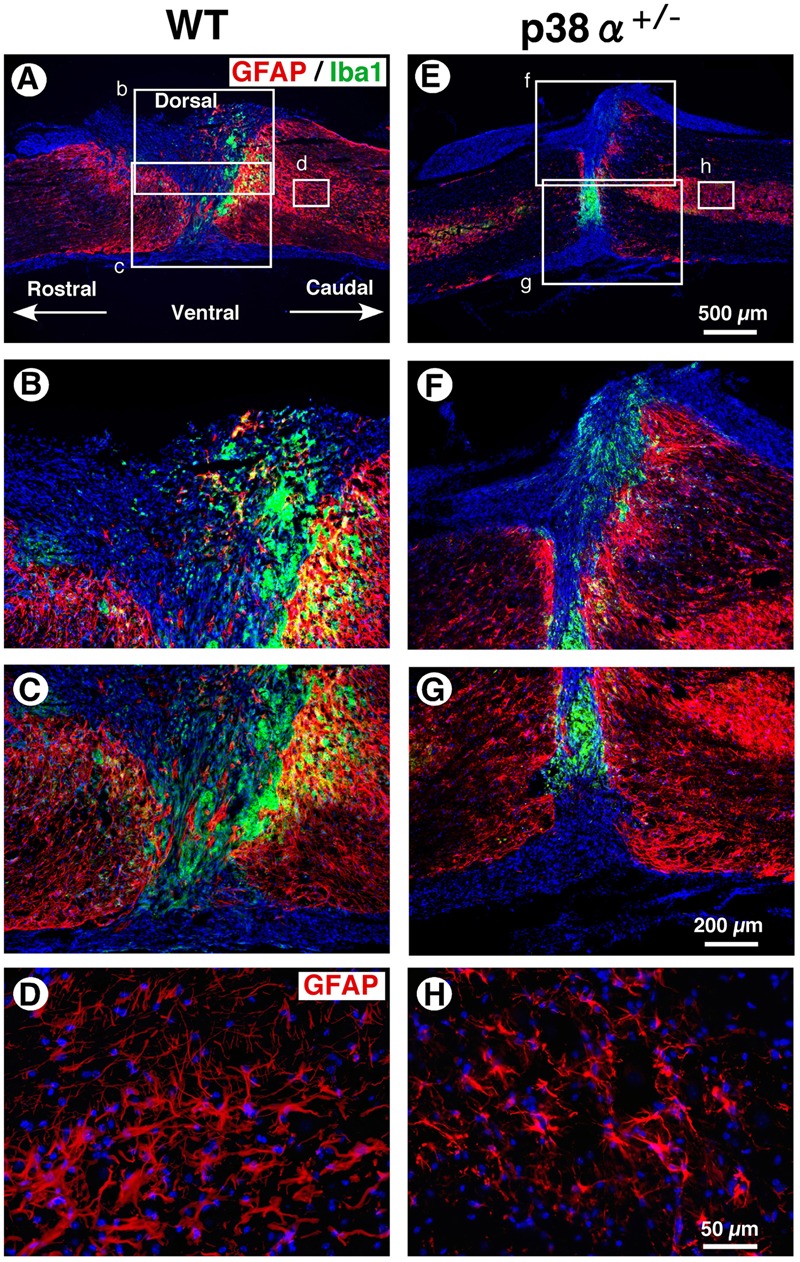
**Astrocytic scar formation and neuroinflammation-associated cells at 2 weeks post-injury. (A,E)** Representative images of sagittal sections showing GFAP^+^ reactive astrocytes and Iba1^+^ cells at 2 weeks post-injury. **(B,C,F,G)** Higher magnification images from the boxed area, b, c, f and g in **(A)** and **(E)**. Iba1^+^ cells were compacted to the lesion center between rostrocaudal reactive astrocytes, which was more apparent in the SC of p38α^+/-^ mice than that of WT mice. **(D,H)** Higher magnification images of GFAP^+^ reactive astrocytes from the boxed area, d (in **A**) and h (in **E**). GFAP^+^ reactive astrocytes at 1 mm caudal to the lesion epicenter decreased in p38α^+/-^ mice compared with WT mice.

In a great numbers of studies, glial scar by reactive astrocytes has been regarded as physical barriers to successful axon regeneration (Silver and Miller, 2004). In contrast, recent studies have provided the possibility that reactive astrogliosis have beneficial effects in axonal regeneration via forming the astrocyte bridge, a scaffold for axonal growth (Anderson et al., 2016; Mokalled et al., 2016). On the other hand, activated microglias and macrophages markedly infiltrate into the lesion of CNS injury, the spread degree of which is closely associated with substantial tissue repair and functional restoration (Penkowa et al., 1999). Thus, the cellular formation of GFAP^+^ astrocytes and Iba1^+^ microglias/macrophages between p38α^+/-^ mice and WT mice was examined at 2 wpi. As shown in **Figure 5**, astrocytic scar formation was observed in the epicenter of the two genotypes, which was more massive in WT mice (B and C) than p38α^+/-^ mice (F and G). Likewise, a larger numbers of reactive astrocytes with hypertrophied somas and long processes were observed in WT mice (D) than p38α^+/-^ mice (H). These phenomena were supported by a previous study that astrogliosis after CNS damage might be attenuated in astrocyte-specific p38α-knockout mice (Roy Choudhury et al., 2014). On the other hand, Iba1^+^ microglias/macrophages were accumulated more compactly between rostrocaudal GFAP^+^ reactive astrocytes in p38α^+/-^ mice than WT mice (**Figures 5B,C,F,G**). Likewise, the distance between rostrocaudal glial scars was shorter in p38α^+/-^ mice than WT mice (**Figures 5A,E**), indicating that contraction of lesion area was enhanced in p38α^+/-^ mice compared with WT mice. It has been reported that the Stat3-upregulated migratory activity of reactive astrocytes to seclude inflammatory cells enhances contraction of lesion area and functional restoration after SCI (Okada et al., 2006). Moreover, a loss of function of p38α in astrocyte negatively affects its cellular migration activity (Roy Choudhury et al., 2014). Thus, more typical compaction of microglias/macrophages associated with smaller wound area in p38α^+/-^ mice compared with WT mice is of interest. Probably, at least the less infiltration of inflammatory cells related to expression of proinflammatory cytokines (**Figure 3**) may positively affect the wound healing process in p38α^+/-^ mice even though astrogliosis-forming activity is moderate compared with WT mice. In addition, it was also provided the possibility that the contribution of NSCs to wound healing/tissue regeneration process after SCI might be potentiated in p38α^+/-^ mice.

**FIGURE 6:**
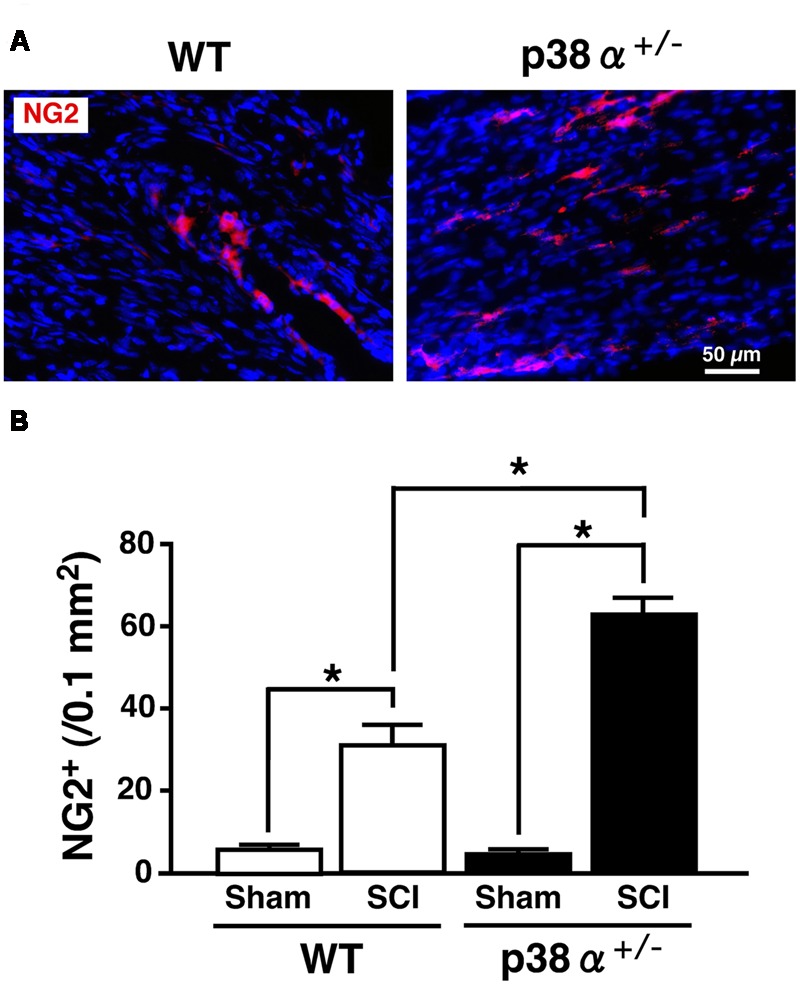
**Change in numbers of NG2^+^ cells in the white matter at 2 weeks after SCI. (A)** Typical profile of NG2^+^ cells in the lesion epicenter of the two genotypes at 2 weeks post-injury. **(B)** NG2^+^ cells in the injured site within 500 μm rostrocaudal of the lesion epicenter in the SCI group were increased in the two genotypes at 1 week post-injury, the number of which was significantly higher in p38α^+/-^ mice (closed bars) than WT mice (open bars). Data are shown as mean ± SEM (*n* = 4–5). ^∗^*P* < 0.05 (ANOVA followed by Tukey’s test).

Multipotent NSCs are defined as cells that can self-renew and differentiate into the three neuronal lineages, neuron, astrocyte, and oligodendrocyte (Gage, 2000). In the SC, NSCs known as ependymal cells in the central canal proliferate rapidly after SCI and differentiate into more than half the astrocytes in the glial scar and a small amount of oligodendrocytes (Barnabé-Heider et al., 2010). Likewise, *in vitro*, NSCs derived from adult WT mice easily and exclusively differentiate into astrocyte with repeating cell passages without appropriate neurotrophic factors (Seaberg and van der Kooy, 2002; Bull and Bartlett, 2005). Hence, the low differentiation capacity of adult NSC for neural and oligodendrocytic lineages can limit the recovery from SCI. We previously reported that NSCs in the adult hippocampus of p38α^+/-^ mice have much higher self-renewal activity and neural differentiation capacity compared with those of WT mice (Yoshioka et al., 2015). These findings tempted us to think that activity of progenitor cells for oligodendrocyte and neuron might be upregulated in p38α^+/-^ mice under the tissue regeneration process after SCI. To elucidate this point, we observed cell populations of oligodendrocyte precursor cells (OPCs) and neural progenitor cells in the lesion epicenter of SC at 2 wpi. As we expected, the number of NG2^+^ OPCs in the white matter of the lesion increased in the two genotypes at 2 wpi, which was significantly higher in p38α^+/-^ mice than in WT mice (**Figure 6**). NG2^+^ OPCs into the injured SC can enhance remyelination of spared axons and improve functional recovery after SCI (Whittaker et al., 2012). Thus, in conjunction with the results shown in **Figures 2B,C**, the increase in OPCs in p38α^+/-^ mice after SCI may contribute to the efficiency of remyelination under the tissue regeneration process. We also determined that NG2^+^ cells were more accumulated along the laceration/epicenter in p38α^+/-^ mice compared with WT mice (data not shown). This accumulation was very similar to typical histopathological findings in CNS damages including SCI (Tan et al., 2005). NG2 is a member of chondroitin sulphate proteoglycans (CSPGs) generally known to be repulsive to growing axons. However, NG2 also called CSPG4 has been recently recognized as a promoting molecule for axonal growth and regeneration (Yang et al., 2006). In fact, it has been demonstrated that astrocytes forming a bridge across a scar after SCI highly express CSPG4/NG2 (Anderson et al., 2016). Likewise, NG2^+^ OPCs provide an adhesive substrate for axonal growth by forming a bridge after SCI (Busch et al., 2010). Although we could not detected the astrocytic bridge between rostrocaudal glial scars in the two genotypes (**Figure 5**), the fact that accumulation of NG2^+^ OPCs along the laceration/epicenter was more apparent in p38α^+/-^ mice compared with WT mice might affect the efficiency of axonal regeneration between the two genotypes. In addition to the upregulation of OPC recruitment in p38α^+/-^ mice, an increase in DCX^+^ neural progenitor cells in the gray matter of the lesion was bigger in p38α^+/-^ mice than in WT mice at 2 wpi. Some of the DCX^+^ neural progenitor cells also expressed Nestin and were observed in area proximal to the lesion epicenter in the two genotypes. Notably, DCX^+^Nestin^+^ cells were also detected within the epicenter in case of p38α^+/-^ mice but not WT mice (Supplementary Figure 2). DCX^+^Nestin^+^ cells have been identified as resident multipotent NSCs in the SC meninges and at least contribute to parenchymal reaction following SCI (Decimo et al., 2011). Although it is still unclear whether the DCX^+^Nestin^+^ cells recruited into the lesion contribute to neural regeneration, the finding that recruitment of neuroblasts and resident NSCs to the lesion was upregulated in p38α^+/-^ mice may affect the subsequent tissue regeneration in concert with the enhanced recruitment of OPCs.

Together, a single copy disruption of *p38*α *gene* affected the tissue degeneration and regeneration processes and improved the functional recovery from SCI. However, the question arose as to whether the SCI-augmented p38 activation was inhibited in the SC of p38α^+/-^ mice. Although SCI did not affect each expression level of p38α in the two genotypes, the SCI-augmented p38 activation was less in p38α^+/-^ mice compared with WT mice (Supplementary Figure 3). Therefore, we finally evaluated the effect of a p38 inhibitor on the functional recovery from SCI.

### A p38 Inhibitor Improved Locomotor Recovery after SCI

**FIGURE 7:**
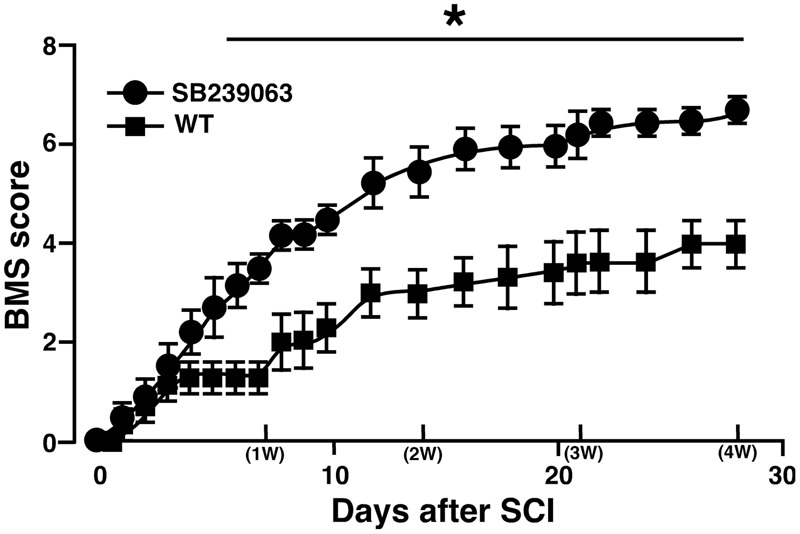
**Effect of SB239063 on hindlimb locomotor behavior after SCI.** WT mice with SCI received oral administration of vehicles (*filled squares*) and SB239063 (10 mg/kg per day, *filled circles*) at 1, 2, and 3 dpi. Functional recovery from SCI was better in mice with SB239063 treatment than those with vehicle administration. Data are shown as mean ± SEM (*n* = 6). ^∗^*P* < 0.05, a significant difference between the two groups by Mann–Whitney *U* test for unpaired values at each time point.

Unlike the previous studies on the association between SCI and a p38 inhibitor (Horiuchi et al., 2003; Stirling et al., 2008), we used SB239063 as a p38 inhibitor that is an orally active and transferable across the blood-cerebrospinal fluid barrier. In the preliminary study, we confirmed that an oral administration of SB239063 (10 mg/kg body weight) could efficiently inhibit the SCI-induced p38α activity (Supplementary Figure 4). Then, based on a previous report that the strong activation of p38 MAPK in the injured SC increases from 12 h to 3 days (Song et al., 2013), we orally administrated SB239063 to WT mice (10 mg/kg per day) at 1, 2, and 3 dpi. As we expected, the BMS score was significantly higher in the SB239063-treated group compared with the vehicle group over a time period ranging from 6 to 28 dpi (**Figure 7**), indicating that a pharmacological inhibition of p38α also improved the recovery of hindlimb behavior after SCI.

Our present result that SB239063 is effective on the functional recovery after lateral hemisection of the SC can be supported by the previous study employing a mild contusion model of SCI by Horiuchi et al. (2003). On the other hand, another study demonstrated that a p38α inhibitor failed to improve functional outcome after SCI with moderate contusion (Stirling et al., 2008). Currently, the discrepancy in efficacy of a p38α inhibitor for SCI between them is difficult to explain. Regardless of the type of SCI model, however, the intensity of SC damage may influence the beneficial effect of a p38α inhibitor. In addition, the route and schedule of administration with a p38α inhibitor also affect its efficacy. As a next step, to investigate whether our protocol of SB239063 administration is effective in a moderate contusion model of SCI is needed.

## Conclusion

A single copy disruption of *p38*α *gene* inhibited the tissue degenerative events such as leukocytic infiltration, expression of cytokines/chemokines and apoptosis of oligodendrocyte and neuron and enhanced the tissue regenerative events such as compaction of microglias/macrophages, recruitment of OPCs and NSC and axonal regrowth and remyelination, which resulted in a better functional recovery from SCI. We also demonstrated that a pharmacological inhibition of p38α could recapitulate the better functional recovery from SCI observed in p38α^+/-^ mice. Our present study clearly suggests that p38α contributes to the pathogenesis of SCI and propose that an orally active p38α inhibitor, SB239063 may have therapeutic benefits for the treatment of SCI.

## Author Contributions

HU, KoT, YK developed the concept and designed the experiments. HU, YN, KeT, and YK performed experiments. KY and KS performed statistical analysis. TS, MHag, and MHat gave conceptual advice. HU and YK wrote the paper. All authors discussed the results and implications and commented on the manuscript at all stages.

## Conflict of Interest Statement

The authors declare that the research was conducted in the absence of any commercial or financial relationships that could be construed as a potential conflict of interest.

## References

[B1] Anderson M. A. Burda J. E. Ren Y. Ao Y. O’Shea T. M. Kawaguchi R. (2016). Astrocyte scar formation aids central nervous system axon regeneration. *Nature* 532 195–200. 10.1038/nature17623 27027288 PMC5243141

[B2] Barnabé-Heider F. Göritz C. Sabelström H. Takebayashi H. Pfrieger F. W. Meletis K. (2010). Origin of new glial cells in intact and injured adult spinal cord. *Cell Stem Cell* 7 470–482. 10.1016/j.stem.2010.07.014 20887953

[B3] Basso D. M. Fisher L. C. Anderson A. J. Jakeman L. B. McTigue D. M. Popovich P. G. (2006). Basso Mouse Scale for locomotion detects differences in recovery after spinal cord injury in five common mouse strains. *J. Neurotrauma* 23 635–659. 10.1089/neu.2006.23.635 16689667

[B4] Beck K. D. Nguyen H. X. Galvan M. D. Salazar D. L. Woodruff T. M. Anderson A. J. (2010). Quantitative analysis of cellular inflammation after traumatic spinal cord injury: evidence for a multiphasic inflammatory response in the acute to chronic environment. *Brain* 133 433–447. 10.1093/brain/awp322 20085927 PMC2858013

[B5] Becker D. Sadowsky C. L. McDonald J. W. (2003). Restoring function after spinal cord injury. *Neurologist* 9 1–15. 10.1097/01.nrl.0000038587.58012.05 12801427

[B6] Brunn A. Utermöhlen O. Carstov M. Ruiz M. S. Miletic H. Schlüter D. (2008). CD4 T cells mediate axonal damage and spinal cord motor neuron apoptosis in murine p0106-125-induced experimental autoimmune neuritis. *Am. J. Pathol* 173 93–105. 10.2353/ajpath.2008.071101 18535178 PMC2438288

[B7] Bull N. D. Bartlett P. F. (2005). The adult mouse hippocampal progenitor is neurogenic but not a stem cell. *J. Neurosci.* 25 10815–10821. 10.1523/JNEUROSCI.3249-05.2005 16306394 PMC6725873

[B8] Busch S. A. Horn K. P. Cuascut F. X. Hawthorne A. L. Bai L. Miller R. H. (2010). Adult NG2^+^ cells are permissive to neurite outgrowth and stabilize sensory axons during macrophage-induced axonal dieback after spinal cord injury. *J. Neurosci.* 30 255–265. 10.1523/JNEUROSCI.3705-09.2010 20053907 PMC2823089

[B9] Decimo I. Bifari F. Rodriguez F. J. Malpeli G. Dolci S. Lavarini V. (2011). Nestin- and doublecortin-positive cells reside in adult spinal cord meninges and participate in injury-induced parenchymal reaction. *Stem Cells* 29 2062–2076. 10.1002/stem.766 22038821 PMC3468739

[B10] Dong H. Fazzaro A. Xiang C. Korsmeyer S. J. Jacquin M. F. McDonald J. W. (2003). Enhanced oligodendrocyte survival after spinal cord injury in Bax-deficient mice and mice with delayed Wallerian degeneration. *J. Neurosci.* 23 8682–8691. 14507967 10.1523/JNEUROSCI.23-25-08682.2003 PMC6740425

[B11] Donnelly D. J. Popovich P. G. (2008). Inflammation and its role in neuroprotection, axonal regeneration and functional recovery after spinal cord injury. *Exp. Neurol.* 209 378–388. 10.1016/j.expneurol.2007.06.009 17662717 PMC2692462

[B12] Emery E. Aldana P. Bunge M. B. Puckett W. Srinivasan A. Keane R. W. (1998). Apoptosis after traumatic human spinal cord injury. *J. Neurosurg.* 89 911–920. 10.3171/jns.1998.89.6.0911 9833815

[B13] Gage F. H. (2000). Mammalian neural stem cells. *Science* 287 1433–1438. 10.1126/science.287.5457.1433 10688783

[B14] Gensel J. C. Wang Y. Guan Z. Beckwith K. A. Braun K. J. Wei P. (2015). Toll-like Receptors and Dectin-1, a C-Type Lectin Receptor, Trigger Divergent Functions in CNS Macrophages. *J. Neurosci.* 35 9966–9976. 10.1523/JNEUROSCI.0337-15.2015 26156997 PMC4495245

[B15] Horiuchi H. Ogata T. Morino T. Chuai M. Yamamoto H. (2003). Continuous intrathecal infusion of SB203580, a selective inhibitor of p38 mitogen-activated protein kinase, reduces the damage of hind-limb function after thoracic spinal cord injury in rat. *Neurosci. Res.* 47 209–217. 10.1016/S0168-0102(03)00216-5 14512145

[B16] Horner P. J. Gage F. H. (2000). Regenerating the damaged central nervous system. *Nature* 407 963–970. 10.1038/35039559 11069169

[B17] Jaerve A. Müller H. W. (2012). Chemokines in CNS injury and repair. *Cell Tissue Res.* 349 229–248. 10.1007/s00441-012-1427-3 22700007

[B18] Kang Y. J. Chen J. Otsuka M. Mols J. Ren S. Wang Y. (2008). Macrophage deletion of p38alpha partially impairs lipopolysaccharide-induced cellular activation. *J. Immunol.* 180 5075–5082. 10.4049/jimmunol.180.7.5075 18354233

[B19] Kigerl K. A. Lai W. Rivest S. Hart R. P. Satoskar A. R. Popovich P. G. (2007). Toll-like receptor (TLR)-2 and TLR-4 regulate inflammation, gliosis, and myelin sparing after spinal cord injury. *J. Neurochem.* 102 37–50. 10.1111/j.1471-4159.2007.04524.x 17403033

[B20] Kumar S. Boehm J. Lee J. C. (2003). p38 MAP kinases: key signalling molecules as therapeutic targets for inflammatory diseases. *Nat. Rev. Drug Discov.* 2 717–726. 10.1038/nrd1177 12951578

[B21] Li G. L. Farooque M. Holtz A. Olsson Y. (1999). Apoptosis of oligodendrocytes occurs for long distances away from the primary injury after compression trauma to rat spinal cord. *Acta Neuropathol.* 98 473–480. 10.1007/s004010051112 10541870

[B22] Lu P. Blesch A. Graham L. Wang Y. Samara R. Banos K. (2012). Motor axonal regeneration after partial and complete spinal cord transection. *J. Neurosci.* 32 8208–8218. 10.1523/JNEUROSCI.0308-12.2012 22699902 PMC3407545

[B23] Maruyama M. Sudo T. Kasuya Y. Shiga T. Hu B. Osada H. (2000). Immunolocalization of p38 MAP kinase in mouse brain. *Brain Res.* 887 350–358. 10.1016/S0006-8993(00)03063-8 11134625

[B24] Matsuo Y. Amano S. Furuya M. Namiki K. Sakurai K. Nishiyama M. (2006). Involvement of p38alpha mitogen-activated protein kinase in lung metastasis of tumor cells. *J. Biol. Chem.* 281 36767–36775. 10.1074/jbc.M604371200 17028194

[B25] Mokalled M. H. Patra C. Dickson A. L. Endo T. Stainier D. Y. Poss K. D. (2016). Injury-induced ctgfa directs glial bridging and spinal cord regeneration in zebrafish. *Science* 354 630–634. 10.1126/science.aaf2679 27811277 PMC5114142

[B26] Mostacada K. Oliveira F. L. Villa-Verde D. M. Martinez A. M. (2015). Lack of galectin-3 improves the functional outcome and tissue sparing by modulating inflammatory response after a compressive spinal cord injury. *Exp. Neurol.* 271 390–400. 10.1016/j.expneurol.2015.07.006 26183316

[B27] Namiki K. Matsunaga H. Yoshioka K. Tanaka K. Murata K. Ishida J. (2012). Mechanism for p38α-mediated experimental autoimmune encephalomyelitis. *J. Biol. Chem.* 287 24228–24238. 10.1074/jbc.M111.338541 22637476 PMC3397849

[B28] Namiki K. Nakamura A. Furuya M. Mizuhashi S. Matsuo Y. Tokuhara N. (2007). Involvement of p38alpha in kainate-induced seizure and neuronal cell damage. *J. Recept. Signal Transduct. Res.* 27 99–111. 10.1080/1079989070135785 17613723

[B29] Neirinckx V. Coste C. Franzen R. Gothot A. Rogister B. Wislet S. (2014). Neutrophil contribution to spinal cord injury and repair. *J Neuroinflammation* 11 150 10.1186/s12974-014-0150-2 PMC4174328 25163400

[B30] Noble L. J. Donovan F. Igarashi T. Goussev S. Werb Z. (2002). Matrix metalloproteinases limit functional recovery after spinal cord injury by modulation of early vascular events. *J. Neurosci.* 22 7526–7535. 12196576 10.1523/JNEUROSCI.22-17-07526.2002 PMC2792199

[B31] Noubade R. Krementsov D. N. Del Rio R. Thornton T. Nagaleekar V. Saligrama N. (2011). Activation of p38 MAPK in CD4 T cells controls IL-17 production and autoimmune encephalomyelitis. *Blood* 118 3290–3300. 10.1182/blood-2011-02-336552 21791428 PMC3179398

[B32] Okada S. Nakamura M. Katoh H. Miyao T. Shimazaki T. Ishii K. (2006). Conditional ablation of Stat3 or Socs3 discloses a dual role for reactive astrocytes after spinal cord injury. *Nat. Med.* 12 829–834. 10.1038/nm1425 16783372

[B33] O’Keefe S. J. Mudgett J. S. Cupo S. Parsons J. N. Chartrain N. A. Fitzgerald C. (2007). Chemical genetics define the roles of p38alpha and p38beta in acute and chronic inflammation. *J. Biol. Chem.* 282 34663–34671. 10.1074/jbc.M704236200 17855341

[B34] Onifer S. M. Rabchevsky A. G. Scheff S. W. (2007). Rat models of traumatic spinal cord injury to assess motor recovery. *ILAR J.* 48 385–395. 10.1093/ilar.48.4.385 17712224

[B35] Ousman S. S. David S. (2001). MIP-1alpha, MCP-1, GM-CSF, and TNF-alpha control the immune cell response that mediates rapid phagocytosis of myelin from the adult mouse spinal cord. *J. Neurosci.* 21 4649–4656. 11425892 10.1523/JNEUROSCI.21-13-04649.2001 PMC6762369

[B36] Oyinbo C. A. (2011). Secondary injury mechanisms in traumatic spinal cord injury: a nugget of this multiply cascade. *Acta Neurobiol. Exp.* 71 281–299. 10.55782/ane-2011-1848 21731081

[B37] Penkowa M. Carrasco J. Giralt M. Moos T. Hidalgo J. (1999). CNS wound healing is severely depressed in metallothionein I- and II-deficient mice. *J. Neurosci.* 19 2535–2545. 10087067 10.1523/JNEUROSCI.19-07-02535.1999 PMC6786080

[B38] Risco A. del Fresno C. Mambol A. Alsina-Beauchamp D. MacKenzie K. F. Yang H. T. (2012). p38γ and p38δ kinases regulate the Toll-like receptor 4 (TLR4)-induced cytokine production by controlling ERK1/2 protein kinase pathway activation. *Proc. Natl. Acad. Sci. U.S.A.* 109 11200–11205. 10.1073/pnas.1207290109 22733747 PMC3396476

[B39] Roy Choudhury G. Ryou M. G. Poteet E. Wen Y. He R. Sun F. (2014). Involvement of p38 MAPK in reactive astrogliosis induced by ischemic stroke. *Brain Res.* 1551 45–58. 10.1016/j.brainres.2014.01.013 24440774 PMC3987968

[B40] Seaberg R. M. van der Kooy D. (2002). Adult rodent neurogenic regions: the ventricular subependyma contains neural stem cells, but the dentate gyrus contains restricted progenitors. *J. Neurosci.* 22 1784–1793. 11880507 10.1523/JNEUROSCI.22-05-01784.2002 PMC6758891

[B41] Silver J. Miller J. H. (2004). Regeneration beyond the glial scar. *Nat. Rev. Neurosci.* 5 146–156. 10.1038/nrn1326 14735117

[B42] Song Y. Liu J. Zhang F. Zhang J. Shi T. Zeng Z. (2013). Antioxidant effect of quercetin against acute spinal cord injury in rats and its correlation with the p38MAPK/iNOS signaling pathway. *Life Sci.* 92 1215–1221. 10.1016/j.lfs.2013.05.007 23688865

[B43] Stirling D. P. Cummins K. Mishra M. Teo W. Yong V. W. Stys P. (2014). Toll-like receptor 2-mediated alternative activation of microglia is protective after spinal cord injury. *Brain.* 137 707–723. 10.1093/brain/awt341 24369381

[B44] Stirling D. P. Liu J. Plunet W. Steeves J. D. Tetzlaff W. (2008). SB203580, a p38 mitogen-activated protein kinase inhibitor, fails to improve functional outcome following a moderate spinal cord injury in rat. *Neuroscience* 155 128–137. 10.1016/j.neuroscience.2008.05.007 18562123

[B45] Takanami-Ohnishi Y. Amano S. Kimura S. Asada S. Utani A. Maruyama M. (2002). Essential role of p38 Mitogen-activated protein kinase in contact hypersensitivity. *J. Biol. Chem.* 277 37896–37903. 10.1074/jbc.M207326200 12138127

[B46] Tamura K. Sudo T. Senftleben U. Dadak A. M. Johnson R. Karin M. (2000). Requirement for p38alpha in erythropoietin expression: a role for stress kinases in erythropoiesis. *Cell* 102 221–231. 10.1016/S0092-8674(00)00027-1 10943842

[B47] Tan A. M. Zhang W. Levine J. M. (2005). NG2: a component of the glial scar that inhibits axon growth. *J. Anat.* 207 717–725. 10.1111/j.1469-7580.2005.00452.x 16367799 PMC1571583

[B48] Ventura J. J. Tenbaum S. Perdiguero E. Huth M. Guerra C. Barbacid M. (2007). p38alpha MAP kinase is essential in lung stem and progenitor cell proliferation and differentiation. *Nat. Genet.* 39 750–758. 10.1038/ng2037 17468755

[B49] Whittaker M. T. Zai L. J. Lee H. J. Pajoohesh-Ganji A. Wu J. Sharp A. (2012). GGF2 (Nrg1-β3) treatment enhances NG2^+^ cell response and improves functional recovery after spinal cord injury. *Glia* 60 281–294. 10.1002/glia.21262 22042562

[B50] Yang Z. Suzuki R. Daniels S. B. Brunquell C. B. Sala C. J. Nishiyama A. (2006). NG2 glial cells provide a favorable substrate for growing axons. *J. Neurosci.* 26 3829–3839. 10.1523/JNEUROSCI.4247-05.2006 16597737 PMC6674118

[B51] Yoshioka K. Namiki K. Sudo T. Kasuya Y. (2015). p38α controls self-renewal and fate decision of neurosphere-forming cells in adult hippocampus. *FEBS Open Bio* 5 437–444. 10.1016/j.fob.2015.05.001 PMC4472823 26101740

[B52] Zhang H. Trivedi A. Lee J. U. Lohela M. Lee S. M. Fandel T. M. (2011). Matrix metalloproteinase-9 and stromal cell-derived factor-1 act synergistically to support migration of blood-borne monocytes into the injured spinal cord. *J. Neurosci.* 31 15894–15903. 10.1523/JNEUROSCI.3943-11.2011 22049432 PMC3265932

[B53] Zhou X. He X. Ren Y. (2014). Function of microglia and macrophages in secondary damage after spinal cord injury. *Neural Regen. Res.* 9 1787–1795. 10.4103/1673-5374.143423 25422640 PMC4239768

