# Supplementary material for: Genetic and Pharmacological Inhibition of p38α Improves Locomotor Recovery after Spinal Cord Injury
Source: Front Pharmacol. 2017 Feb 17;8:72. doi: 10.3389/fphar.2017.00072 (PMC5313485; doi:10.3389/fphar.2017.00072)
Supplement: Supplementary file 1 [file Data_Sheet_1.PDF]

Supplementary Figure 1

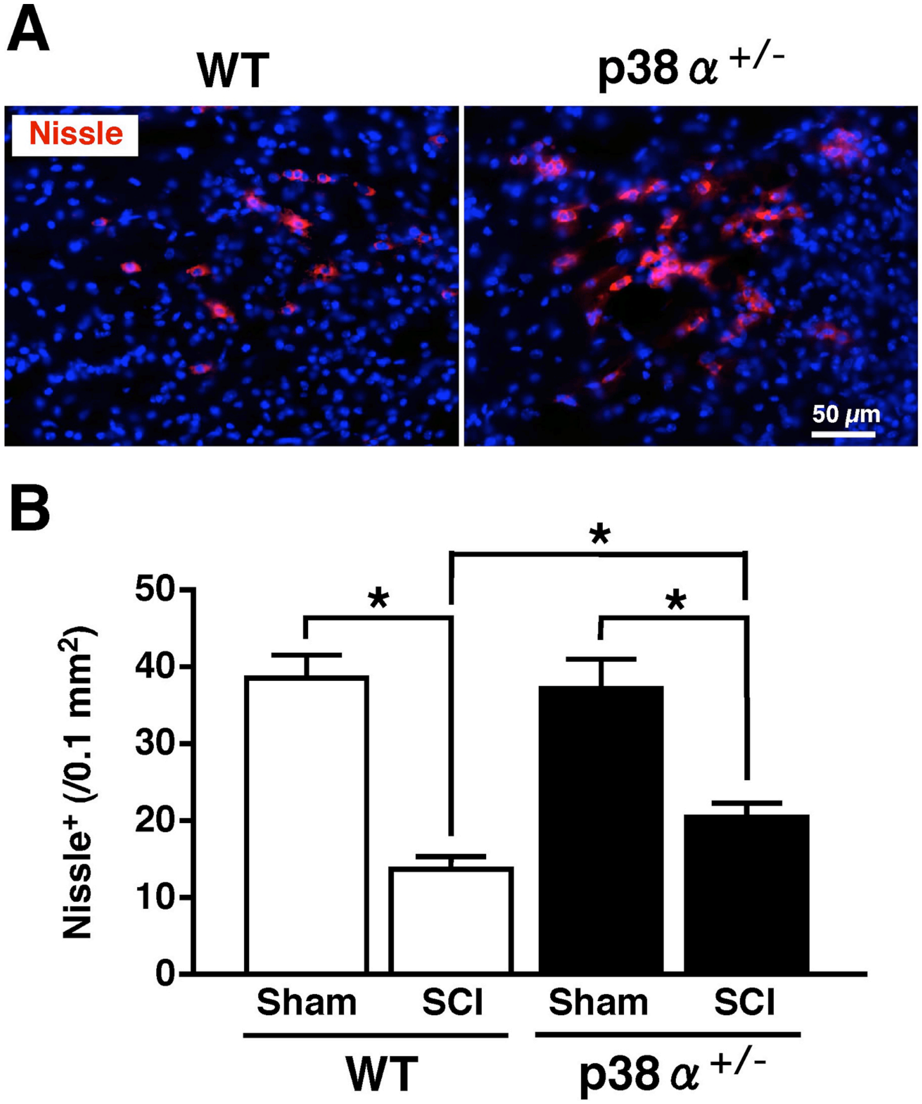

Changes in numbers of Nissle$^+$ neural cells in the grey matter of spinal cord after SCI. ***A)*** Typical profiles of Nissle$^+$ neural cells in the lesion epicenter of WT and p38α$^{+/-}$ mice at 1 week after SCI are shown. ***B)*** Quantitative evaluation of Nissle$^+$ neural cells between the two genotypes was performed with or without SCI. Data are shown as mean ± S.E.M. (n=4-5). *$P < 0.05$ (ANOVA followed by Tukey's test). Sagittal sections (20 μm in thickness) of the spinal cords were prepared from the two genotypes at 1 week after operation (sham-operation or hemisection) and subjected to staining with Nissle reagent (BrainStain Imaging Kit, Thermo Fisher Scientific). Nuclei were stained with 4',6-Diamidino-2-phenylindole, dihydrochloride (DAPI). The lesioned area between 500 μm rostral and 500 μm caudal of the lesion epicenter in the SCI group and the corresponding area in the sham-operated group were observed by a fluorescent microscopy (AXIO Imager A2, Carl Zeiss).
