# Supplementary material for: Genetic and Pharmacological Inhibition of p38α Improves Locomotor Recovery after Spinal Cord Injury
Source: Front Pharmacol. 2017 Feb 17;8:72. doi: 10.3389/fphar.2017.00072 (PMC5313485; doi:10.3389/fphar.2017.00072)
Supplement: Supplementary file 2 [file Data_Sheet_2.PDF]

Supplementary Figure 2

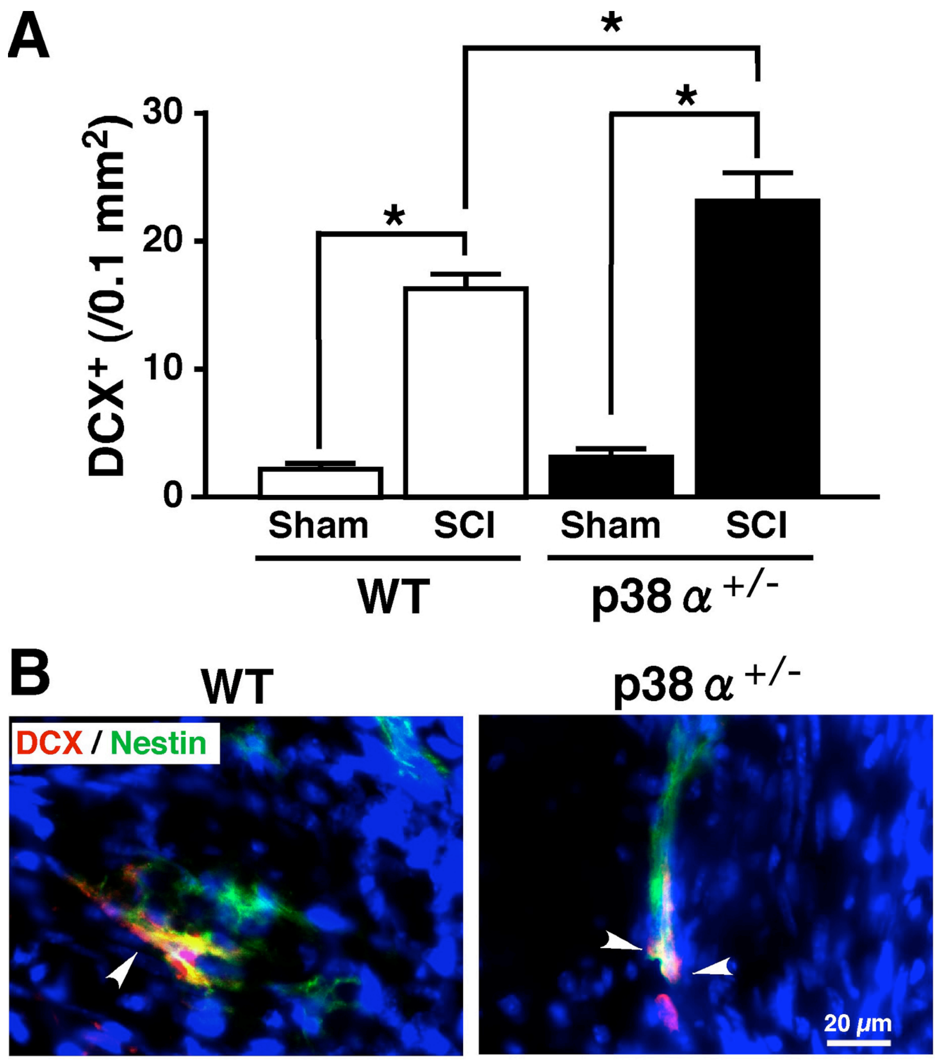

Changes in numbers of doublecortin (DCX)$^+$ neural progenitor cells in the grey matter of spinal cord after SCI. ***A)*** DCX$^+$ neural progenitor cells increased 2 weeks after SCI, which was significantly greater in p38α$^{+/-}$ mice compared with WT mice. The area between 500 μm rostral and 500 μm caudal of the lesion epicenter in the SCI group and the corresponding area in the sham-operated group were observed. Data are shown as mean ± S.E.M. (n=4-5). *$P < 0.05$ (ANOVA followed by Tukey's test). ***B)*** Some of the DCX$^+$ neural progenitor cells appeared to be Nestin$^+$ simultaneously. DCX$^+$Nestin$^+$ cells were also observed in area proximal to the lesion epicenter of the two genotypes. Notably, DCX$^+$Nestin$^+$ cells were observed within the epicenter in not WT mice but p38α$^{+/-}$ mice. Sagittal sections (20 μm in thickness) of the spinal cords were prepared from the two genotypes at 2 weeks after operation (sham-operation or hemisection) and subjected to immunofluorescent study with anti-DCX (C-18, Santa Cruz Biotech.) and anti-Nestin (Sigma-Aldrich) antibodies. Nuclei were stained with DAPI.
