# Supplementary material for: Genetic and Pharmacological Inhibition of p38α Improves Locomotor Recovery after Spinal Cord Injury
Source: Front Pharmacol. 2017 Feb 17;8:72. doi: 10.3389/fphar.2017.00072 (PMC5313485; doi:10.3389/fphar.2017.00072)
Supplement: Supplementary file 3 [file Data_Sheet_3.PDF]

Supplementary Figure 3

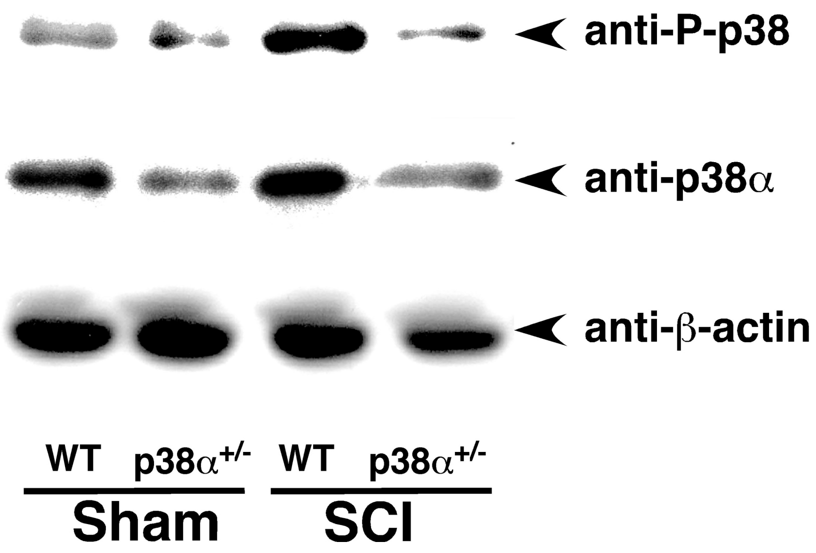

Effects of SCI on protein level and activation of p38α in the two genotypes. Protein samples used in Western blot array (Fig. 3C) were subjected to Western blot analyses with anti-phospho p38, anti-p38α (Cell Signaling Technology, Danvers, MA) and anti-β-actin (Sigma-Aldrich). Similar results were obtained from three independent experiments.
