# Supplementary material for: Genetic and Pharmacological Inhibition of p38α Improves Locomotor Recovery after Spinal Cord Injury
Source: Front Pharmacol. 2017 Feb 17;8:72. doi: 10.3389/fphar.2017.00072 (PMC5313485; doi:10.3389/fphar.2017.00072)
Supplement: Supplementary file 4 [file Data_Sheet_4.PDF]

Supplementary Fig. 4

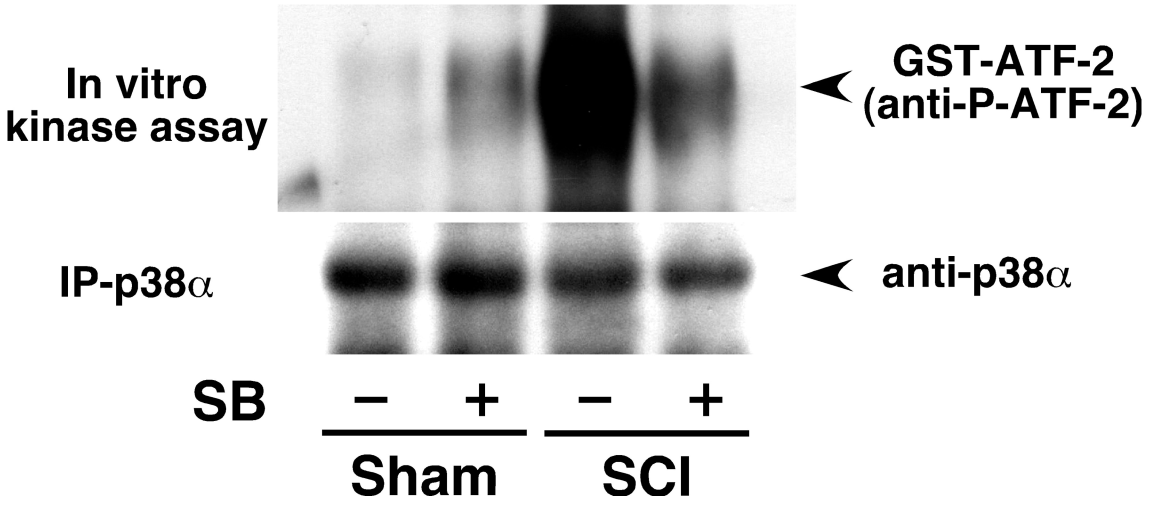

Inhibitory effect of SB239063 on the SCI-induced p38α activity. Mice were orally administered with SB239063 (10 mg/Kg) or vehicle (acidified 0.5% tragacanth) 24 h after the operation (Sham-operation or SCI). Then, protein samples were prepared from the SCs 36 h after the operation. Each protein sample (200 μg) was subjected to the immunoprecipitation with anti-p38α antibody (Ref.: Maruyama M, et al. 2000) in combination with protein A/G-agarose (Calbiochem, San Diego, CA). After washing with a kinase buffer, the immunoprecipitates were mixed with 200 μM ATP and 2 μg GST-ATF-2 fusion protein as a substrate in 30 μl of kinase buffer and incubated for 45 min at 30°C. The kinase reaction was terminated by adding an appropriate volume of SDS sample buffer. The phosphorylation of ATF-2 was determined by Western blot analysis with anti-phospho-ATF-2 (Thr71) antibody (Cell Signaling Technology). As an internal control, the amount of immunoprecipitated p38α was determined by Western blot analysis with anti-p38α antibody (Cell Signaling Technology). Similar results were obtained from two independent experiments.
